# Towards Defining Molecular Determinants Recognized by Adaptive Immunity in Allergic Disease: An Inventory of the Available Data

**DOI:** 10.1155/2010/628026

**Published:** 2011-02-13

**Authors:** Kerrie Vaughan, Jason Greenbaum, Yohan Kim, Randi Vita, Jo Chung, Bjoern Peters, David Broide, Richard Goodman, Howard Grey, Alessandro Sette

**Affiliations:** ^1^The Immune Epitope Database (IEDB), La Jolla Institute for Allergy & Immunology (LIAI), La Jolla, CA 92037, USA; ^2^School of Medicine, Allergy and Immunology, University of California, San Diego, La Jolla, CA 92093, USA; ^3^Food Allergy Research and Resource Program, University of Nebraska-Lincoln, Lincoln, NE 68583, USA

## Abstract

Adaptive immune responses associated with allergic reactions recognize antigens from a broad spectrum of plants and animals. Herein a meta-analysis was performed on allergy-related data from the immune epitope database (IEDB) to provide a current inventory and highlight knowledge gaps and areas for future work. The analysis identified over 4,500 allergy-related epitopes derived from 270 different allergens. Overall, the distribution of the data followed expectations based on the nature of allergic responses. Namely, the majority of epitopes were defined for B cells/antibodies and IgE-mediated reactivity, and relatively fewer T-cell epitopes, mostly CD4^+^/class II. Interestingly, the majority of food allergen epitopes were B-cells epitopes whereas a fairly even number of B- and T-cell epitopes were defined for airborne allergens. In addition, epitopes from nonhumans hosts were mostly T-cell epitopes. Overall, coverage of known allergens is sparse with data available for only ~17% of all allergens listed by the IUIS database. Thus, further research would be required to provide a more balanced representation across different allergen categories. Furthermore, inclusion of nonpeptidic epitopes in the IEDB also allows for inventory and analysis of immunological data associated with drug and contact allergen epitopes. Finally, our analysis also underscores that only a handful of epitopes have thus far been investigated for their immunotherapeutic potential.

## 1. Introduction

It is estimated that 50 million people in the US are affected by airborne allergens, including approximately 35 million affected by upper respiratory allergies (allergic rhinitis, hay fever and pollinosis) [1], and 16 million affected by asthma [2, 3]. The cost of allergies in the US (treatment and loss of work) is estimated to be more than $18 billion per year [4]. Food allergies, representing the second largest category after respiratory allergies, are thought to affect 6–8% of children and nearly 4% of adults. In the US, there are ~30,000 episodes of food-induced anaphylaxis, associated with 100–200 deaths per year [5, 6]. Finally, skin contact allergies and allergies to insect venoms also occur with significant incidence and are thus important component of allergic diseases in humans. These figures underscore the growing societal impact of allergy-related disease both in terms of human suffering as well as annual cost burden. 

The immunological basis of allergy-related disease is universally recognized. At the level of adaptive immunity, the recognition of specific allergens by antibodies and T cells plays major roles both as effectors and regulators of allergic diseases. Several bioinformatics resources, cataloging and describing allergen protein sequences, are available to the scientific community such as the *Allergome*, which provides information on allergenic molecules causing IgE-mediated disease, and *Allergen.org*, which is the official site for the systematic allergen nomenclature approved by the World Health Organization and International Union of Immunological Societies (WHO/IUIS) Allergen Nomenclature Sub-committee]. However, until recently, a resource describing the actual epitopes (defined as the molecular structures coming in contact with antibodies or T-cell receptors), and the immunological events associate with epitope recognition, was not available. 

The immune epitope database (IEDB) was created, to provide the scientific community with a repository of freely accessible immune epitope data (http://www.immuneepitope.org/). It contains data curated from published literature, -submitted by the NIAID's high-throughput epitope discovery projects, and imported from other databases. The database contains antibody and T-cell data for human, nonhuman primate, and rodent hosts, as well as a number of other animal species, and targets epitopes associated with infectious diseases, autoimmunity, transplantation, and allergy. Data related to both peptidic and non-peptidic epitopes are curated within the IEDB.

Recently, curation of allergy-related references was completed, and as a result, close to 10,000 records, capturing 4,800 different epitopes, and the biological assays associated with their recognition have become available within the IEDB. By analogy with what was done in the case of epitopes associated with influenza, tuberculosis, malaria, and other diseases [7–9], herein we report the results of an in-depth analysis of all published immune epitope data related to Allergy. This analysis provides an inventory of epitope structures and associated data in order to enhance basic research and facilitate the development of therapeutics, as well as diagnostics. The analysis also identified certain knowledge gaps and points to potential areas for future study. 

## 2. Methods

### 2.1. Data Inclusion Criteria

This analysis includes data for antibody and T-cell epitopes associated with allergic disease in human and nonhuman (animal models) hosts. To identify within the IEDB the subset of data which is allergy related, we followed the process described in more detail by Davies et al. [10]. More specifically, we define herein allergy-related data on the basis of the source from which the epitope is derived (known allergen), and also on the basis of the type of response and/or clinical presentation. Accordingly, we included IgE-mediated, type I (immediate) hypersensitivity, atopic, “allergen-sensitization,” exposure-based asthma, allergic rhinitis, pollinosis, contact dermatitis, atopic dermatitis, documented anaphylaxis, and all data from allergy-related animal models.

 The allergy-related epitopes represent both peptidic as well as non-peptidic structures from a wide range of sources, including pollens, dust mites, molds, dander and foods, nonprotein moieties of plants (carbohydrates), as well as drugs, haptens, metals, and chemical substances from occupational exposures. The curated data was obtained using a variety of different assays, such as ELISA, Western blot, proliferation assays, surface plasmon resonance (SPR), radio immunoassay (RIA), and X-ray crystallography, and describes epitope-related reactivity such as histamine release, hypersensitivity (PCA), delayed-type hypersensitivity (DTH), and immunotherapy assays. 

All of the data described herein were captured directly from the peer-reviewed literature (PubMed) by Ph.D. level scientists or through direct submission to the IEDB by research groups. Antibody and T-cell epitope definitions (length and mass restrictions) as well as IEDB inclusion criteria can be found at http://tools.immuneepitope.org/wiki/index.php/Main_Page. For the purpose of this report, the total number of epitopes reported in each case represents the total number of unique molecular structures experimentally shown to react with a B- cell or T-cell receptor (no predictions included). The IEDB captures these structures as they are defined in the literature and thus includes data describing structures categorized as minimal/optimal epitopes (11–15 resides), larger less well-defined regions (20–50 residues), and key residues identified as being involved in binding (1-2 residues). 

### 2.2. Analysis Approach

The entirety of the allergy-related data identified as described above was first inventoried to identify the total number of structures (positive and negative epitopes), their chemical nature (peptidic or non-peptidic), the total number of antibody/B-cell versus T-cell epitopes, as well as the effector cell phenotype or antibody isotype, and finally the total number of peer-reviewed references from which the data were derived. The second step involved investigating the distribution of epitopes among hosts: those epitopes defined in humans versus those identified using nonhuman animal models of allergy. In each case, the inventory of epitopes per host species included a breakdown according to reactivity: B cell (linear or conformational) or T cell (CD4, CD8 or unspecified).

Following the initial inventory, the data were categorized according to the following established allergy categories: food, airborne (respiratory), contact, drug, and allergies to biting insects. This categorization was based on the *allergen and genus species* of the organism from which the epitope was derived. These main categories were then further parsed into subcategories on the basis of taxonomic origins (plant, animal or fungus) and included a subcategory for the most commonly encountered species in that main category.

The individual compounds representing drugs/pharmaceuticals were parsed into 21 subcategories on the basis of its chemical type (e.g., beta-lactam antibiotic) or by the way the compound is used to treat a particular condition (e.g., muscle relaxant). Contact allergen data were also further parsed into subcategories based on their species of origin (plants), chemical type (metals, model haptens), or mode of exposure (chemical agents from occupational exposure). 

### 2.3. Computational Methods

The allergy-related data extracted from the IEDB (http://www.immuneepitope.org/) was stored in a MySQL database. The use of MySQL allows for the tailoring of database schema to the specific analysis and to keep the data synchronized with updates of the IEDB data production database. Data were periodically checked against the IEDB webpage using simple or advanced query interfaces for consistency and accuracy. Results from each query were exported as Excel files and further analyzed in that format. Tables and figures were generated from Excel. Data exclusions included structures for which only MHC binding data were available, as well as those instances in which the epitope was simultaneously used as both immunogen and assay antigen. 

## 3. Results

### 3.1. Data Overview

An overview of all allergy-related data captured by our analysis is provided in Tables 1 and 2. Consistent with the importance of immunoglobulin-related responses as effectors of allergy responses, the majority of epitopes (both peptidic and non-peptidic) were defined for antibody responses, including both linear (~3,000) and conformational (or discontinuous) determinants (peptidic only) (Table 1). A total of 2,205 IgE epitopes were reported for all allergens, and less numerous other reactivities related to total IgG followed distantly by IgG1 IgG4, IgM, IgA, IgG2b, IgG3, IgG2a, and IgG2c (Table 2). As can be seen, the majority of antibody determinants were defined in humans. In animal models of disease, not only relatively fewer epitopes were defined, but only about 10% of them are epitopes recognized by IgE. This highlights a crucial knowledge gap and suggests that more research could be directed at the definition of the epitopes recognized by IgE in animal models of allergy.

A relatively smaller number of T-cell epitopes have been identified (1,646 epitopes) (Table 1). Of the T-cell epitopes defined in both peptidic and non-peptidic allergens, CD4^+^/Class II epitopes were most numerous, and far fewer CD8^+^/Class I epitopes were reported. Given their potential role in contact dermatitis and other delayed-type hypersensitivity reactions, it is likely that more effort could be devoted to the definition of class I epitopes. Supplementary Figure 1 (see Figure s1 in supplementary material available on line at doi: 10.1155/2007/628026) provides a response summary for all epitope data.

The host distribution of epitopes can be found in Table 3. Not surprisingly, the vast majority of epitopes were defined in humans. However, epitopes were also described for monkeys, pigs, dogs, rabbits, guinea pigs, rats, and mice. Of the nonhuman species, epitopes defined in mice represented the second largest group. Within epitopes defined in mice, more than 30 different strains were represented (data not shown). BALB/c predominated, followed distantly by C57BL/6 and C3H/He. Data from human HLA transgenic strains (HLA-A, DR4, DQ6, DQ8, and DR3-DQ2) were also reported. Table 1 also describes a breakdown of epitope numbers categorized as related to food allergies, airborne or respiratory allergies, allergies to stinging insects, drug allergies, and contact allergies. Food allergens represent by far the largest group of data in the IEDB. There are currently 2,322 (53%) B- and T-cell epitopes identified from this group. After food allergens, epitopes defined for aeroallergens represent the second largest group, accounting for 40% of the records. To date, the database contains 125 antibody and T-cell epitopes related to the venom of stinging insects, which make up 3% of the epitope total. Drug allergies account for ~2% of epitopes. Contact allergies manifested through the skin account for ~2% of allergy epitopes. The following sections describe each epitope category in more detail. 

### 3.2. Food Allergies

These include both peptidic and non-peptidic determinants derived from both plants and animals. The data have been parsed into three broad categories; most common food allergen sources, other plant, and other animal species (Table 4). Peanut (*Arachis hypogaea*) allergens which comprise nearly 40% of the total plant allergen epitopes. Epitopes described for food allergens derived from animals fall into six taxonomic categories. These include mammals (human and cow milk, beef, beef gelatin), bony fish (cod), bird (chicken eggs), mollusks (abalone and snails), crustaceans (shrimp and prawns), and nematodes (fish meat parasite). By far, the largest number of epitopes has been identified for allergens related to cow's milk allergy, followed by epitopes defined from eggs, representing 70% and 20% of the total, respectively. Non-peptidic food epitopes reported to date are comprised of carbohydrates derived from peanuts, sugar beets, celery, and sea squirt (see also supplemental Table 6). 

According to the CDC [11], allergens derived from milk, eggs, peanuts, tree nuts, fish, shellfish, soy, and wheat account for 90% of all food allergies. The epitope data in general reflect this distribution. However, fewer epitopes were identified from fish (only 10 epitopes for one species) and shellfish other than shrimp. Conversely, there was a surprising number of epitopes described from fruit allergens, namely peaches, apples, and bananas. This observation may reflect the involvement of these species in the oral allergy syndrome (OAS) or pollen-food allergy and cross-reactions between foods (fruits, nuts) and inhaled allergens [12–15]. 

### 3.3. Airborne Allergies

Epitopes defined for aeroallergens represent the second largest group within the IEDB, accounting for 40% of the records, including peptidic and non-peptidic determinants derived from plants, animals, fungal allergens, and some industrial chemical agents. Here, the data was parsed into the categories of most common airborne sources, other plant, fungal, and animal species (Table 5). Epitopes identified from pellitory pollen, as well as those from birch and Japanese cedar pollen, are numerous. Epitopes reported for grass pollens come primarily from Timothy grass, ryegrass species, and Kentucky blue grass.

In the taxonomic grouping representing fungi, which includes yeasts and molds, epitopes identified in antigens from *Aspergillus* species dominate (70%). Finally, epitopes derived from aeroallergens from animals fall into three broad taxonomic categories: insects (cockroach and midge), arachnids (house dust mite, Storage mite, and Fodder mite), and mammals (cat, dog, horse, cow, rat, and mouse). Among the insects, epitopes derived from the midge are the most numerous, and within the Arachnid class, European house dust mites are the most heavily studied. 

Three non-peptidic determinants were described (see supplemental Table 6). These included *α*-L-Fuc-(1->3)-[*α*-D-Man-(1->3)-[*α*-D-Man-(1->6)]-[*β*-D-Xyl-(1->2)]-*β*-D-Man-(1->4)-*β*-D-GlcNAc-(1->4)]-D-GlcNAc from cedar pollen, D-glucopyranuronic acid from the fungus *Trichosporon cutaneum,* and mono-*β*-arabinofuranose from mugwort pollen. In addition, a number of non-peptidic chemicals were identified, including compounds causing respiratory symptoms following occupational exposure; toluene 2,4-diisocyanate (TDI), toluene meta-diisocyanate, 4-tolyl isocyanate, diphenylmethane-4,4′-diisocyanate, hexamethylene diisocyanate, phenyl isocyanate, phthalic anhydride, and trimellityl group (data not shown).

Here again, the epitope data reflects the overall trends related to airborne allergy. Grass, tree, and weed pollen epitopes represent the majority of the data (~60%), followed by pet dander and house dust mite allergens. These findings are consistent with the overall prevalence of hay fever and/or allergic rhinitis in the general population, affecting some 18 million people annually [16]. Perhaps somewhat unexpected, was the fairly low number of epitopes defined for cat allergens. Interestingly, the majority of food allergen epitopes were B-cells epitopes (86%) whereas a fairly even number of B (43%) and T-cell (57%) epitopes were defined for airborne allergens (data not shown). 

### 3.4. Allergies to Stinging Insects

To date, the IEDB contains 125 antibody and T-cell epitopes related to the venom of stinging insects (Table 6).These include honeybee (*Apis mellifera*), bald-faced hornets (*Dolichovespula maculate*), black-bellied hornets (*Vespa basalis*), common wasps (*Vespula vulgaris*), and ants (*Myrmecia pilosula*). Of these, epitopes derived from honeybees represent the largest portion, followed by wasps and bald-faced hornets. Surprisingly, only 2 B-cell epitopes for ant venom, 1 B-cell epitope for Black-faced hornets, and no antibody determinants for wasp venom were defined. There are also two non-peptidic determinants for antibody reactivity to honey bee venom. These include *α*-1,3-fucose and an N(4)-[*α*-L-fucosyl-(1->3)-N-acetyl-4-O-glycosyl-D-glucosaminyl]-L-asparagine residue (see supplemental Table 6). 

### 3.5. Drug Allergies

**Figure 1:**
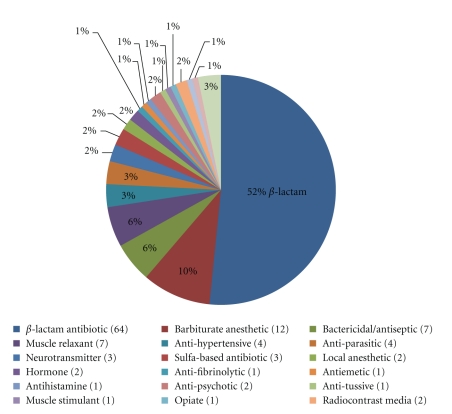
Drug allergens by functional category. Determinants identified under this category have been broadly classified into 21 groups according to their overall biological functional. The chart presents these data as percentages with the total number of unique assays in parentheses.

The IEDB currently contains curated data relating to immunological reactions to more than 90 different drugs associated with allergic disease. In most cases, the authors do not identify the exact reactive moiety of these non-peptidic chemical entities because the assays are carried out using the intact drug. These drugs can be further classified into 21 categories based primarily on biological function and structure (Figure 1). These include beta-lactam antibiotics (the penicillins), barbiturate anesthetics, bactericidal/antimicrobial, muscle relaxants, antihypertensive, antiparasitic drugs, neurotransmitters, sulfa-based antibiotics, local anesthetics, hormones, antifibrinolytics, antiemetics, antihistamines, antipsychotics, antitussives, muscle stimulants, opiates, radiocontrast media, spermicides, and a vasoactive agonist. Antibiotics as a whole comprise nearly half (49%) of the reported drug allergens, with the vast majority of which are beta-lactam antibiotics. 

### 3.6. Contact Allergies

**Figure 2:**
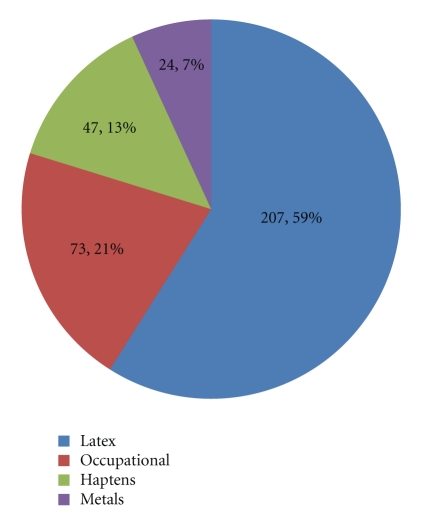
Categories of contact allergen epitopes. The chart provides a broad overview of the contact allergen epitope distribution.

Thus far, more than 80 contact allergens have been captured by the IEDB, as summarized in Figure 2. Epitopes identified from latex-allergic individuals represent the largest number of contact allergen determinants, making up 59% of the total. A total of reported 207 latex epitopes include both linear and nonlinear antibody epitopes, as well as T-cell epitopes, primarily of the CD4^+^/class II phenotype.

Three additional categories of contact allergens include *non-peptidic* entities such as metals, industrial chemicals encountered by way of occupational exposure, and model haptens. A total of seven different metals described as associated with allergic contact dermatitis include beryllium (beryllium sulfate tetrahydrate, beryllium sulfate), chromium (chromium trichloride), cobalt (cobalt dichloride), copper (copper sulfate, copper chloride), nickel (nickel chloride, nickel sulfate) palladium (palladium chloride), and zinc chloride. Of these, no single metal entity stands predominates, and as a group metals comprise only 7% of the contact allergens. Beryllium, chromium, zinc chloride, and cobalt are most often encountered in the industrial/manufacturing setting, whereas nickel, copper, and palladium allergies are most frequently associated with jewelry. Furthermore, the IEDB contains curated data relating to more than 70 compounds utilized in the manufacture of cosmetics, dyes, and certain constituents of manufacturing. A very large number of curated assays relate to model haptens, which include skin sensitizers such as trinitrophenyl (TNP), dinitrophenyl (DNP), 1-fluoro-2,4-dinitrobenzene (DNFB), and dinitrochlorobenzene (DNCB). These compounds have been used classically to define mechanisms of type IV contact hypersensitivity. Of these, DNCB appears to have received the greatest focus. A detailed list of all contact allergens can be found in supplemental Table 1. 

### 3.7. Epitope Distribution by Allergen

As a further evaluation, we determined the relative epitope distribution by allergen for each source species (supplementary Tables 2–5). The total number of epitopes described per allergen varies greatly, and well-known allergens (e.g., Ara h 1, Bet v 1, or Phl p 1) tended to have greater numbers of defined epitopes compared to other allergens from the same organism (e.g., seed storage protein SSP2, Bet v 2, Bet v 4, Phl p 2, or Phl p 11). Similarly, the total number of T-cell versus B-cell epitopes varied greatly, with the vast majority of allergens heavily weighted toward one or the other phenotype and few having a relative balance of defined B and T epitopes (data not shown). 

Next, we analyzed the extent to which the allergens comprising the epitope-related data represent all known allergens, as listed by the *Allergen.org* resource, the official site for the systematic allergen nomenclature (Linnean system) that is approved by the World Health Organization and International Union of Immunological Societies (WHO/IUIS) Allergen Nomenclature Sub-committee. This site maintains a list of all currently known (described) allergens derived from plant, animal, and fungal species. We found that total number of allergens from which epitope data have been described varies from one allergen source to another. In some instances, epitope data is comprehensive, showing epitope data for all allergens identified by the IUIS list for a given species (e.g., 9/9 Phl p allergens for timothy grass). However in other cases, allergen distribution is low, showing only a few of the known allergens (e.g., 6/29 Der p and Der f allergens for house dust mite), whereas other species have intermediate distribution (e.g., 4/6 Lol p allergens from rye grass) (Table 7). Furthermore, when we compared the total number of allergens in the IUIS that match the allergy-related species reported in the IEDB, we find that ~40% of the IUIS-designated allergens are represented in the epitope data (115 out of 297). However, for an additional 380 known IUIS allergens, no match could be found between the species in the IUIS and the species described in the papers in the scientific literature describing specific epitopes. Many of these include organisms from known genera, but with as yet nonlisted species, as well as other nomenclature inconsistencies. These results suggest that more efforts can be devoted to reconciling the origin of allergen-derived data. 

### 3.8. Epitopes Associated with Clinical Disease or Disease Models

Isolated epitopes can be utilized to induce or modulate allergic reactions in animal models. The use of synthetic epitopes to modulate allergic reactions has also been proposed and tested in a limited number of clinical trials [17, 18]. Indeed, the epitopes defined in the course study of human allergic conditions may enable the investigation of their potential in the immunotherapeutic setting. 

To inventory which epitopes had been tested in these settings, we queried for antibody and T-cell epitopes that were tested either *in vivo* for their ability to decrease allergic reactivity *in vivo* (as measure by the reduction of symptoms) and for those that were shown to decrease *in vitro* markers of allergic disease. This is done by selecting all B-cell or T-cell contexts designated in the IEDB as assay type equals “Reduction of Disease after Treatment” (B cell) or “Treatment” (T cell). Here, the assay type assigned by the IEDB indicates the nature of the immune response, and the details of the type of assay used (lung function, DTH, PCA, etc.) can be found within the curated data from the assay comments field.  Table 8 shows the PubMed identification, epitope name, epitope sequence, the host, the type of response, and allergy model classification for peptidic epitopes identified from the data as having a positive effect on disease *in vivo* or on markers of disease as measured *in vitro*. 

## 4. Discussion

The analysis presented herein identified over 4,500 allergy-related epitopes derived from 270 different allergens. Protein allergens were categorized according to their source organism, which included plants, animals, insects, parasites, and fungi. Non-peptidic allergens were categorized into four groups including drugs and biologicals, industrial compounds, or those related to occupational exposure, metals, model haptens, and carbohydrates from plants.

Overall, the distribution of the data follows expectations based on the nature of adaptive responses involved in allergy. Namely, the vast majority of allergy epitopes were defined for B cells/antibodies (and in these records, IgE-mediated reactivity figured prominently), and relatively fewer T-cell epitopes (mostly defined as CD4^+^/class II, with very few being defined for CD8^+^/class I). Likewise, most of the records related to the study of allergic reactions in humans, and fewer epitopes defined for mice and occasional epitopes defined for other hosts such as monkeys, pigs, dogs, rabbits, guinea pigs, and rats. The majority of peptidic epitopes were defined for foods (cow's milk, wheat, peanuts) and plants (tree and grass pollens), while the majority of non-peptidic epitopes defined for drugs and biologicals (antibiotics). 

Interestingly, the vast majority of food allergen-related epitopes were described for B-cells, whereas a fairly even number of B- and T-cell epitopes were defined for airborne allergens. It is not clear why this is the case but may have to do with historical analysis of allergies to foods such as milk, peanuts, and eggs which represent a large portion of that data. The distribution of epitopes varies greatly between allergen and species. This observation suggests that definition of T-cell epitopes involved in food allergies is lacking and could be the focus of further experimental investigations. 

Another unexpected finding of our analysis was that the epitopes defined in hosts other than humans were mostly T-cell epitopes, and far fewer antibody epitopes were defined. While it is surprising that so little of the nonhuman antibody responses are allergy-specific IgE; this may point to an important area for experimental investigation, to provide investigators with animal models faithfully reproducing human allergic reactions.

The current analysis also revealed that coverage of known human allergen by epitope definition studies is very sparse. The overall completeness of the epitope-specific allergy data with respect to known allergens on a species basis is about 40%. Furthermore, epitope data is available for only ~17% of all allergens listed by IUIS. For certain species, the majority (if not all) of the known allergens have epitope-related data (e.g., timothy grass allergens), while other species have epitope data from only a small number of known allergens (e.g., apple).

The recent completion of curation of non-peptidic allergy-related epitopes in the IEDB allows a first time inventory and assessment of important drug and contact allergens. The integration within the IEDB of representation and search capabilities based on the chemical entity of biological interest (ChEBI) (http://www.ebi.ac.uk/chebi/) database will further enable the scientific community to quickly retrieve and analyze the immunological data associated with these important classes of allergens. 

Finally, our analysis also inventoried which epitopes have been used to actively induce allergic disease in animal models or to modulate disease. Only a handful of epitopes have been investigated for their immunotherapeutic potential. If the promising results from human clinical trials were to be verified in later phase trails, we anticipate that the data cataloged within the IEDB might provide a wealth of leads for therapeutic intervention regimens. 

## Supplementary Material

Supplementary materials contain the following:
Supplemental Figure 1: Overall response summary.
Supplementary Table 1: Contact allergens.
Supplementary Table 2: Epitope distribution in different food allergens.
Supplementary Table 3: Epitope distribution in different airborne allergens.
Supplementary Table 4: Epitope distribution among stinging insects.
Supplementary Table 5: Epitope distribution for latex allergens.
Supplementary Table 6: Carbohydrate epitopes associated with allergic reactions.

## Figures and Tables

**Table 1:** Overview of allergy epitope data included in the IEDB.

Category	Total number
References	678
Epitopes (positive structures)	4,800
Negative structures	4,137
Antibody/B cell	3,194
Linear natural peptides	2,730
Linear analogs/no natural source	163
Discontinuous determinants	65
T cell	1,646
CD4^+^/Class II	1,530
CD8^+^/Class I	17
Unspecified	99
Unique allergens (peptidic/nonpeptidic)	550
Source species	88
Food allergy epitopes	2,322 (53%)
Airborne allergy epitopes	1,750 (40%)
Stinging insects epitopes	127 (3%)
Drug allergy epitopes	106 (~2%)
Contact allergy epitopes	82 (~2%)

**Table 2:** Antibody isotype associated with epitope reactivity.

Antibody isotypes	Number of reactive epitopes		
	Overall	Human	Nonhuman
Allergy-specific IgE	2,205	2,135	70
*Other antibody reactivity*			
IgG, unspecified	495	192	303
IgG1	132	0	132
IgG4	72	72	0
IgM	50	32	18
IgG2b	10	0	10
IgA	5	0	5
IgG3	5	2	3
IgG2a	2	0	2
IgG2c	1	0	1

**Table 3:** Host distribution for peptidic and non-peptidic epitopes.

Allergic host	All T cell	CD4, class II	CD8, class I	All B cell	Linear B cell	Nonlinear	Overall
**Peptidic epitopes**							
Human	1,483	1,406	9	2,590	2,541	49	4,073
*Animal models*							
Japanese macaque	0	0	0	2	2	0	2
Pig	0	0	0	5	5	0	5
Dog	23	23	0	0	0	0	23
Rabbit	0	0	0	337	337	0	337
Rat	1	1	0	6	6	0	7
Mouse	304	271	6	388	357	31	692

**Nonpeptidic epitopes**							
Human	50	42	1	72	—	—	122
*Animal models*							
Rabbit	0	0	0	36	—	—	36
Guinea pig	8	0	0	12	—	—	20
Rat	2	1	0	2	—	—	4
Mouse	26	5	2	50	—	—	76

**Table 4:** Epitope data related to food allergy. Genus species have been modified to match IUIS usage. Synonyms for querying the IEDB: tomato (Solanum lycopersicum) and brown shrimp is (*Farfantepenaeus aztecus). *

Category	All T cell	CD4/class II	CD8/class I	All B cell	Linear B cell	Non-linear B cell	Total epitopes
Common food allergen sources							
Cow's milk (*Bos domesticus*)	90	90	0	661	659	2	751
Peanut (*Arachis hypogaea*)	26	26	0	337	337	0	363
Egg (*Gallus domesticus*)	75	59	1	150	149	1	225
Common wheat (*Triticum aestivum*)	0	0	0	128	127	1	128
Soybean (*Glycine max*)	1	1	0	71	71	0	72
Hazelnut (*Corylus avellana*)	27	27	0	27	27	0	54
Brown shrimp (*Penaeus aztecus*)	0	0	0	53	53	0	53
Cashew (*Anacardium occidentale*)	0	0	0	27	27	0	27
Common walnut (*Juglans regia*)	0	0	0	27	27	0	27
Sesame seed (*Sesamum indicum*)	0	0	0	11	11	0	11
Baltic cod (*Gadus callarias*)	0	0	0	10	10	0	10
Brazil nut (*Bertholletia excelsa*)	0	0	0	7	7	0	7

Other plant species							
Peach (*Prunus persica*)	43	43	0	15	15	0	58
Banana (*Musa acuminata*)	1	1	0	51	51	0	52
Buckwheat (*Fagopyrum esculentum*)	0	0	0	39	39	0	39
Apple (*Malus x domestica*)	1	1	0	27	24	3	28
Celery (*Apium graveolens*)	14	0	0	0	0	0	14
Muskmelon (*Cucumis melon*)	0	0	0	12	12	0	12
Oriental mustard (*Brassica juncea*)	0	0	0	9	9	0	9
Rice (*Oryza sativa japonica*)	0	0	0	5	5	0	5
American plum (*Prunus armeniaca*)	0	0	0	4	4	0	4
European plum (*Prunus domestica*)	0	0	0	4	4	0	4
Chinese date (*Ziziphus mauritiana*)	0	0	0	4	4	0	4
Sweet cherry (*Prunus avium*)	1	0	0	3	0	3	4
Common oat (*Avena sativa*)	4	4	0	0	0	0	4
Tomato (*Lycopersicum esculentum*)	0	0	0	2	2	0	2
Yellow mustard (*Sinapis alba*)	0	0	0	2	1	1	2
Chinese cucumber (*Trichosanthes kirilowii*)	0	0	0	1	1	0	1
Goat grass (*Aegilops markgrafii*)	1	1	0	0	0	0	1
Naked oats (*Avena nuda*)	1	1	0	0	0	0	1
Mango (*Mangifera indica*)	0	0	0	1	1	0	1

Other animal species							
Beef (*Bos domesticus*)	2	2	0	10	10	0	12
Human breast milk (*H. sapiens*)	0	0	0	6	6	0	6
Cow gelatin (*Bos domesticus*)	0	0	0	3	3	0	3
Horned turban snail (*Turbo cornutus*)	0	0	0	2	2	0	2
Red abalone (*Haliotis rufescens*)	0	0	0	1	0	1	1
Fish nematode (*Anisakis simplex*)	0	0	0	1	1	0	1

**Table 5:** Epitope data related to Airborne/Respiratory Allergy. Genus species have been modified to match IUIS usage. Synonyms for querying the IEDB: Arizona cypress (*Hesperocyparis arizonica). *

Category	All T cell	CD4/class II	CD8/class I	All B cell	Linear B cell	Non-linear B cell	Total epitopes
Common airborne allergen sources							
Birch tree (*Betula verrucosa*)	175	167	0	36	30	6	211
Japanese cedar (*Cryptomeria japonica*)	181	180	0	19	19	0	200
European house dust mite (D. pteronyssinus)	104	87	2	53	39	14	157
Mold (*Aspergillus fumigatus*)	16	16	0	115	111	4	131
Timothy grass (*Phleum pratense*)	72	71	1	49	48	1	121
Perennial ryegrass (*Lolium perenne*)	69	60	0	36	32	4	105
Midge (*Chironomus thummi thummi*)	30	30	0	60	60	0	90
Olive tree (*Olea europaea*)	14	0	0	65	65	0	79
Cat (*Felis catus*)	48	46	0	18	18	0	66
Japanese cypress (*Chamaecyparis obtusa*)	62	60	0	1	1	0	63
Spreading pellitory (*Parietaria judaica*)	1	1	0	61	60	1	62
Kentucky bluegrass (*Poa pratensis*)	18	0	0	34	34	0	52
Cereal rye (*Secale cereale*)	0	0	0	51	51	0	51
Dog (*Canis lupus familiaris*)	50	50	0	0	0	0	50
American house dust mite (*D. farinae*)	36	36	0	12	9	3	48
Mold (Penicillium chrysogenum)	0	0	0	45	44	1	45
Horse (*Equus caballus*)	42	42	0	1	0	1	43
Bermuda grass (*Cynodon dactylon*)	23	23	0	3	3	0	26
Annual ragweed (*Ambrosia artemisiifolia*)	12	12	0	13	13	0	25

Other plant species							
Common wormwood (*Artemisia vulgaris*)	19	19	0	0	0	0	19
Sunflower (*Helianthus annuus*)	0	0	0	18	18	0	18
Common velvet grass (*Holcus lanatus*)	0	0	0	14	14	0	14
Ashe juniper tree (*Juniperus ashei*)	0	0	0	13	13	0	13
Great ragweed (*Ambrosia trifida*)	5	5	0	0	0	0	5
Loblolly pine tree (*Pinus taeda*)	0	0	0	4	4	0	4
Lichwort (*Parietaria officinalis*)	0	0	0	2	2	0	2
Mouse ear cress (*Arabidopsis thaliana*)	2	2	0	0	0	0	2
Queen Anne's Lace (*Daucus carota*)	1	0	0	0	0	0	1
Elegant zinnia (*Zinnia violacea*)	1	1	0	0	0	0	1
Tobacco (*Nicotiana tabacum*)	0	0	0	1	1	0	1
Tall fescue grass (*Festuca arundinacea*)	0	0	0	1	1	0	1
Arizona cypress tree (*Cupressus arizonica*)	0	0	0	1	1	0	1
Formosan juniper tree (*Juniperus formosana*)	0	0	0	1	1	0	1

Other fungal species							
*Alternaria alternata *	0	0	0	5	5	0	5
* Malassezia sympodialis *	0	0	0	1	0	1	1
*Candida albicans *	0	0	0	1	1	0	1
*Paracoccidioides brasiliensis *	1	0	0	0	0	0	1
*Aspergillus restrictus *	0	0	0	1	1	0	1

Other animal species							
Rat (*Rattus norvegicus*)	19	0	0	4	4	0	23
Storage mite (*Blomia tropicalis*)	0	0	0	18	16	2	18
Fodder mite (*Lepidoglyphus destructor*)	10	10	0	5	5	0	15
German cockroach (*Blattella germanica*)	9	9	0	5	3	2	14
Cow dander (*Bos domesticus*)	8	8	0	2	0	2	10
Mayne's house dust mite (*Euroglyphus maynei*)	10	0	0	0	0	0	10
American cockroach (*Periplaneta americana*)	0	0	0	9	8	1	9
Mouse (*Mus musculus*)	4	2	0	4	4	0	8

**Table 6:** Epitope Data Related to Stinging Insects.

Allergen source	All T cell	CD4/class II	CD8/class I	All B cell	Linear B cell	Non-linear B cell	Total epitopes
Allergen source species							
Honey bee (*Apis mellifera*)	48	47	0	7	5	2	55
Jack jumper ant (*Myrmecia pilosula*)	0	0	0	2	2	0	2
Black-bellied hornet (*Vespa basalis*)	0	0	0	1	1	0	1
Common wasp (*Vespula vulgaris*)	36	36	0	0	0	0	36
Bald-faced hornet (*Dolichovespula maculata*)	20	17	0	11	11	0	31

**Table 7:** Summary of Allergen Coverage. This table provides a comparison of the total number of allergens designated by the IUIS and housed within database *Allergen.org* that match the allergy-related species reported in the IEDB.

Allergy category	Allergen.org	IEDB	Percent coverage
Food	86	39	45%
Airborne or Respiratory	184	65	35%
Stinging insects	14	6	43%
Contact	13	5	38%

	297	115	39%

**Table 8:** Epitopes associated with modulation of allergic disease.

Epitope name	Epitope sequence	Host	Response	Allergy model
Cyn d 1 (127–146)	KAGELTLQFRRVKCKYPSGT	Human	T cell, DCP	Bermuda grass pollen
Cyn d 1 (19–38)	LEAKATFYGSNPRGAAPDDH	Human	T cell, DCP	Bermuda grass pollen
Cyn d 1 (154–173)	KGSNDHYLALLVKYAAGDGN	Human	T cell, DCP	Bermuda grass pollen
Cyn d 1 (136–155)	RRVKCKYPSGTKITFHIEKG	Human	T cell, DCP	Bermuda grass pollen
Cyn d 1 (28–47)	SNPRGAAPDDHGGACGYKDV	Human	T cell, DCP	Bermuda grass pollen
Cyn d 1 (82–101)	VECSGEPVLVKITDKNYEHI	Human	T cell, DCP	Bermuda grass pollen
Cyn d 1 (227–246)	VIPANWKPDTVYTSKLQFGA	Human	T cell, DCP	Bermuda grass pollen
Cyn d 1 (91–110)	VKITDKNYEHIAAYHFDLSG	Human	T cell, DCP	Bermuda grass pollen
Cyn d 1 (73–92)	CYEIKCKEPVECSGEPVLVK	Human	T cell, DCP	Bermuda grass pollen
Fel d 1 IPC-2	KALPVVLENARILKNCVDAKMTEEDKE	Human	T cell, LSC, NSC	Cat allergy
Fel d 1 IPC-1	KRDVDLFLTGTPDEYVEQVAQYKALPV	Human	T cell, LSC, NSC	Cat allergy
peptide 4 (P93-110)	TKCYKLEHPVTGCGERTE	Human	T cell, CLPR	Honey bee sting
peptide 1 (P81-98)	YFVGKMYFNLIDTKCYKL	Human	T cell, CLPR	Honey bee sting
Bet v 1	SKEMGETLLRAVESYLLAHSD	Mouse	B cell, AWI	Birch pollen
Der p 1 111–139	FGISNYCQIYPPNANKIREALAQPQRYCR	Mouse	T cell, DTP	European HDM
Der p 1 113–127	ISNYCQIYPPNANKI	Mouse	T cell, DTP	European HDM
Der p I (101–154)	QSCRRPNAQRFGISNYCQIYPPNVNKIREALAQTHSAIAVIIGIKDLDAFRHYD	Mouse	T cell, DTP	European HDM
Der p 1 110–131	RFGISNYCQIYPPNANKIREAL	Mouse	T cell, DTP	European HDM
Der p I 114–129	SNYCQIYPPNANKIR	Mouse	B cell, DTH	European HDM
Der p 2 87–129	DIKYTWNVPKIAPKSENVVVTVKVMGDDGVLACAIATHAKIRD	Mouse	B cell, BPR, AWI	European HDM
Der p I (98–140)	AREQSCRRPNAQRFGISNYCQIYPPNVNKIREALAQTHSAIAV	Mouse	T cell, DTP	European HDM
Api m 4 7–19	KVLTTGLPALISW	Mouse	B cell, IgG1	Honey bee sting
Dol m 5 176–195	IEDNWYTHYLVCNYGPGGND	Mouse	B cell, IgG1	Hornet sting
Dol m 5 41–60	KNEILKRHNDFRQNVAKGLE	Mouse	T cell, DTP	Hornet sting
Dol m 5 141–160	NYKVGLQNSNFRKVGHYTQM	Mouse	T cell, DTP	Hornet sting
Cry j 2 247–258	AEVSYVHVNGAK	Mouse	B cell, CSI	Japanese cedar pollen
Cry j 2 P2 246–259	RAEVSYVHVNGAKF	Mouse	T cell, NS	Japanese cedar pollen
Ole 1 109–130	TVNGTTRTVNPLGFFKKEALPK	Mouse	B cell, AWI	Olive tree pollen
Phl p 5 peptide	YAATVATAPEVKYTVFETALKKAI	Mouse	B cell, AWI	Timothy grass pollen
Phl p 1 peptide	LRSAGELELQFRRVKCKYPEG	Mouse	B cell, AWI	Timothy grass pollen
Bet v 1/Phl p 1/Phl p 5 hybrid	MGETLLRAVESYAGELELQFRRVKCKYTVATAPEVKYTVFETALK	Mouse	B cell, AWI	Tree/Grass pollen
PI-1 IgECH*ε*2 (109-117)	LYCFIYGHI	Mouse	T cell, PCA	Mouse
nOVA 173–196	VLVNAIVFKGLWEKAFKDEDTQAM	Rat	B cell, PCA	Chicken Egg
OVA (257–264)	SIINFEKL	Mouse	T cell, AWI	Asthma
OVA (323–339)	ISQAVHAAHAEINEAGR	Mouse	T cell, AWI	Asthma

HDM: house dust mite. Mouse strains consisted of BALB/c and C57BL/6. Rats were Norway strain. AWI: airway inflammation (histology). NS: nasal symptoms (sneezing and rubbing). CSI: cytokine suppression of allergen-IgE production. BPR: bronchopulmonary resistance. DTH: delayed type hypersensitivity. LSC: lung score. NSC: nasal score. CLPR: cutaneous late-phase reaction. DTP: decrease allergen-specific T cell proliferation. DCP: decreased allergen-specific cytokine production.
