# Supplementary figures and images for: Towards Defining Molecular Determinants Recognized by Adaptive Immunity in Allergic Disease: An Inventory of the Available Data
Source: J Allergy (Cairo). 2011 Feb 13;2010:628026. doi: 10.1155/2010/628026 (PMC3042621; doi:10.1155/2010/628026)

**Supplemental Figure 1. Overall Response Summary**

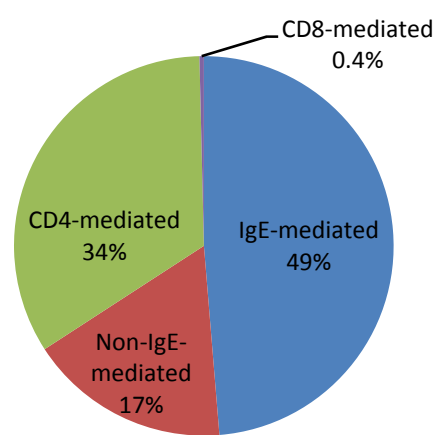

Supplement: Supplementary file 1 — Supplementary materials contain the following: Supplemental Figure 1: Overall response summary. Supplementary Table 1: Contact allergens. Supplementary Table 2: Epitope distribution in different food allergens. Supplementary Table 3: Epitope distribution in different airborne allergens. Supplementary Table 4: Epitope distribution among stinging insects. Supplementary Table 5: Epitope distribution for latex allergens. Supplementary Table 6: Carbohydrate epitopes associated with allergic reactions. [file 628026.f1.pdf]
