# Supplementary material for: Towards Defining Molecular Determinants Recognized by Adaptive Immunity in Allergic Disease: An Inventory of the Available Data
Source: J Allergy (Cairo). 2011 Feb 13;2010:628026. doi: 10.1155/2010/628026 (PMC3042621; doi:10.1155/2010/628026)
Supplement: Supplementary file 2 [file 628026.f2.pdf]

Supplementary Table 1. Contact Allergens

CATEGORY	All T cell	CD4/ Class II	CD8/ Class I	All B cell	Linear B cell	Non-linear B cell	Total Epitopes
**Plants**							
Latex							
Rubber tree (*Hevea brasiliensis*)	43	19	0	164	156	8	**207**
**Metals**							
Nickel	8	4	3	0	NA	NA	**8**
Copper	6	4	2	0	NA	NA	**6**
Cobalt	6	4	1	0	NA	NA	**6**
Zinc dichloride	3	2	1	0	NA	NA	**3**
Beryllium	3	3	0	0	NA	NA	**3**
Palladium	5	5	0	0	NA	NA	**3**
Chromium	2	2	0	0	NA	NA	**2**
**Chemical Agents from Occupational Exposure**			0				
1-fluoro-2,4-dinitrobenzene (DNFB)	6	3	3	1	NA	NA	**7**
1,4-benzoquinone	3	1	1	1	NA	NA	**4**
1,4-phenylenediamine	4	2	1	0	NA	NA	**4**
4-aminophenol	4	1	1	0	NA	NA	**4**
2,4-dinitro-1-thiocyanobenzene	2	1	1	1	NA	NA	**3**
4-methylaminophenol sulfate	3	1	1	0	NA	NA	**3**
ABA diazonium	3	1	2	0	NA	NA	**3**
Alizarin (dye)	0	0	0	1	NA	NA	**3**
Bandrowski's base (dye)	3	2	1	0	NA	NA	**3**
1,3-dicyclohexylcarbodiimide	2	0	0	0	NA	NA	**2**
2,4-dinitrobenzenesulfonate	2	1	1	0	NA	NA	**2**
Aniline	2	2	0	0	NA	NA	**2**
2-aminophenol	2	2	0	0	NA	NA	**2**
1,3-phenylenediamine	2	1	1	0	NA	NA	**2**
2-hydroxypropyl methacrylate	2	1	0	0	NA	NA	**2**
6-(4-hydroxy-5-iodo-3-nitrobenzamido)hexanoic acid	0	0	0	2	NA	NA	**2**
Ethylene glycol dimethacrylate	2	1	0	0	NA	NA	**2**
Methyl methacrylate	2	1	0	0	NA	NA	**2**
N-methylethanolamine	0	0	0	2	NA	NA	**2**
Ethylamine	0	0	0	2	NA	NA	**2**
4,4'-diaminoazobenzene	1	1	0	0	NA	NA	**1**
4,4'-diaminodiphenylmethane	1	1	0	0	NA	NA	**1**
4-bromo-1-hydroxyanthraquinone-2-carboxylic acid	0	0	0	1	NA	NA	**1**
3-aminophenol	0	0	0	1	NA	NA	**1**
6-(4-hydroxy-3-nitrobenzamido)hexanoic acid	0	0	0	1	NA	NA	**1**
1,2-phenylenediamine	1	1	0	0	NA	NA	**1**
Acenaphthene-1,2-dione	0	0	0	1	NA	NA	**1**
2-hydroxyethyl methacrylate	1	1	0	0	NA	NA	**1**
Anilide	0	0	0	1	NA	NA	**1**
4-(2-hydroxy-5-methylphenylazo)acetanilide	1	1	0	0	NA	NA	**1**
anthraflavin	0	0	0	1	NA	NA	**1**
2-[(2-{[2-({3-[2,2,6,6-tetramethyl-1-(ylooxy)piperidin-4-ylamino]phenyl}amino)ethyl]amino}-2-oxoethoxy)acetamido]ethyl 1,2-dipalmitoylglycero-3-phosphate	0	0	0	1	NA	NA	**1**

Bismark Brown Y (dye)	1	1	0	0	NA	NA	**1**
Cetyltrimethylammonium chloride	0	0	0	1	NA	NA	**1**
Coniferol	0	0	0	1	NA	NA	**1**
Ethyl acetate	0	0	0	1	NA	NA	**1**
Nitrosobenzene	1	1	0	0	NA	NA	**1**
Purpurin (dye)	0	0	0	1	NA	NA	**1**
Sodium 2,4-dinitrobenzenesulfonate	0	0	0	1	NA	NA	**1**
**Model Haptens**							
2,4-dinitrophenol (DNP)	10	5	5	3	NA	NA	**13**
Dinitrochlorobenzene (DNCB)	8	3	3	3	NA	NA	**11**
2,4,6-trinitrobenzenesulfonic acid	7	4	3	0	NA	NA	**7**
2,4,6-trinitrophenyl group (TNP)	7	4	3	2	NA	NA	**7**
N(6)-(2,4-dinitrophenyl)-L-lysine	3	1	1	3	NA	NA	**6**
1-chloro-2,4,6-trinitrobenzene	5	2	2	0	NA	NA	**5**
2,4-dinitrobenzenesulfonic acid	4	2	2	0	NA	NA	**4**
(4-hydroxy-5-iodo-3-nitrophenyl)acetyl group	2	0	2	1	NA	NA	**3**
(4-hydroxy-3-nitrophenyl)acetyl group	1	0	0	1	NA	NA	**2**
4,4'-azodibenzenearsonic acid	1	0	0	1	NA	NA	**2**
4-arsonophenyldiazenyl group	1	0	0	1	NA	NA	**2**
N-acetyltyrosine-4-azobenzenearsonate	2	2	0	0	NA	NA	**2**
N-(2,4-dinitro-6-carboxy)phenyl-1.6-diaminohexane	0	0	0	1	NA	NA	**1**
N-(2,4-dinitrophenyl)aminohexanoic acid	0	0	0	1	NA	NA	**1**
4-hydroxy-5-iodo-3-nitrophenylacetyl-O-succinimide ester	1	0	1	0	NA	NA	**1**
Picric acid	0	0	0	1	NA	NA	**1**
1,3-diisopropylcarbodiimide	1	0	0	0	NA	NA	**1**
