# Supplementary material for: Towards Defining Molecular Determinants Recognized by Adaptive Immunity in Allergic Disease: An Inventory of the Available Data
Source: J Allergy (Cairo). 2011 Feb 13;2010:628026. doi: 10.1155/2010/628026 (PMC3042621; doi:10.1155/2010/628026)
Supplement: Supplementary file 3 [file 628026.f3.pdf]

## Supplementary Table 2. Epitope Distribution in Different Food Allergens

| | Name | T cell | B cell | Total Epitopes |
|---|---|---|---|---|
| **Common Food Allergens** | | | | |
| Cow’s milk | Alpha S1 casein | 46 | 168 | **214** |
| | Beta-lactoglobulin | 61 | 122 | **183** |
| | Kappa-casein | 0 | 119 | **119** |
| | Beta-casein | 0 | 111 | **111** |
| | Alpha-S2-casein | 1 | 68 | **69** |
| | Alpha-lactalbumin | 0 | 43 | **43** |
| | Major allergen beta-lactoglobulin | 0 | 34 | **34** |
| Peanut | Allergen Ara h 1, clone P41B precursor | 0 | 191 | **191** |
| | Glycinin | 0 | 71 | **71** |
| | 2S protein 1 | 26 | 11 | **37** |
| | Allergen Arah3/Arah4 | 0 | 35 | **35** |
| | Conglutin-7 precursor | 0 | 30 | **30** |
| | Ara h 2.01 allergen | 0 | 24 | **24** |
| | Allergen II | 0 | 14 | **14** |
| | Seed storage protein SSP2 | 0 | 1 | **1** |
| Chicken egg | Ovomucoid | 55 | 118 | **173** |
| | Ovalbumin | 29 | 28 | **57** |
| | Peptidyl-prolyl cis-trans isomerase B | 0 | 2 | **2** |
| | Ovotransferrin (Allergen Gal d 3) | 1 | 0 | **1** |
| | Serum albumin (Gal d 5) | 0 | 1 | **1** |
| | Serine proteinase inhibitor, clade B | 0 | 1 | **1** |
| Common Wheat | Glutenin | 0 | 52 | **52** |
| | Monomeric alpha-amylase inhibitor | 0 | 51 | **51** |
| | Omega-5 gliadin | 0 | 10 | **10** |
| | Type 1 non-specific lipid transfer protein | 0 | 7 | **7** |
| | Alpha-amylase inhibitor 0.28 | 0 | 5 | **5** |
| | Gamma-gliadin | 0 | 2 | **2** |
| | 27K protein | 0 | 1 | **1** |
| Soybean | Glycinin G2 | 0 | 30 | **30** |
| | P34 probable thiol protease precursor | 0 | 26 | **26** |
| | Beta-conglycinin, alpha chain | 0 | 5 | **5** |
| | Glycinin G1 precursor | 0 | 4 | **4** |
| | Bd 30K (34 kDa maturing seed protein) | 0 | 3 | **3** |
| | Gly m 1 | 0 | 2 | **2** |
| | Profilin-1 | 0 | 1 | **1** |
| | Stress-induced protein SAM22 | 1 | 0 | **1** |
| Common hazel nut | Major allergen Cor a 1.0401 | 27 | 0 | **27** |
| | 11S globulin-like protein | 0 | 25 | **25** |
| | Major pollen allergen Cor a 1 | 0 | 2 | **2** |
| Cashew | 2s albumin | 0 | 16 | **16** |
| | Vicilin-like protein | 0 | 11 | **11** |
| English walnut | Seed storage protein | 0 | 22 | **22** |
| | Albumin seed storage protein | 0 | 5 | **5** |
| Brazil nut | Allergen Ber e 1 | 24 | 5 | **29** |
| | 2S albumin | 0 | 2 | **2** |
| Brown shrimp | Pen a 1 allergen | 0 | 51 | **51** |
| Cod fish | Parvalbumin beta | 0 | 10 | **10** |
| Sesame seed | 2S seed storage protein 1 | 0 | 11 | **11** |
| | | | | |
| **Other Plant Species** | | | | |
| Buckwheat | Fag e 1 | 0 | 26 | **26** |

| | | | | |
|---|---|---|---|---|
| | 13S globulin | 0 | 11 | **11** |
| | 13S globulin seed storage protein 1 | 0 | 2 | **2** |
| Apple | Mal d 3 | 0 | 24 | **24** |
| | Major allergen Mal d 1 | 1 | 3 | **4** |
| Tomato | Probable pectate lyase P59 | 0 | 2 | **2** |
| | Acid beta-fructofuranosidase | 0 | 1 | **1** |
| Rice | Hypothetical protein 115471171 | 0 | 3 | **3** |
| | Hypothetical protein 115482336 | 0 | 2 | **2** |
| Common oat | Avenin precursor (clone pAv122) | 3 | 0 | **3** |
| | Gamma 3 avenin | 1 | 0 | **1** |
| Sweet cherry | Major allergen Pru av 1 | 1 | 3 | **4** |
| Mango | Ripening-related pectate lyase | 0 | 1 | **1** |
| Apricot | Major allergen Pru ar 3 | 0 | 2 | **2** |
| Plum | Major allergen Pru d 3 | 0 | 3 | **3** |
| Indian jujube | Allergen Ziz m 1 | 0 | 4 | **4** |
| Peach | Non-specific lipid transfer protein (Pru p 3) | 43 | 17 | **60** |
| Oriental mustard | Lactoylglutathione lyase | 0 | 9 | **9** |
| Yellow mustard | Allergen Sin a 1 | 0 | 2 | **2** |
| Muskmelon | Profilin | 0 | 12 | **12** |
| Chinese cucumber | Ribosome-inactivating protein | 0 | 1 | **1** |
| Celery | Major allergen Api g 1 | 14 | 0 | **14** |
| Goat grass | HMW glutenin subunit | 1 | 0 | **1** |
| Naked oat | Avenin gamma-3 - small naked oat | 1 | 0 | **1** |
| | | | | |
| **Other Animal Species** | | | | |
| Human Breast milk | Beta-casein precursor | 0 | 6 | **6** |
| Cow gelatin | Collagen, type I, alpha 2 | 0 | 3 | **3** |
| Horned turban snail | Major allergen Tur c1 | 0 | 2 | **2** |
| Red abalone | Tropomyosin | 0 | 1 | **1** |
| Nematode parasite | UA3-recognized allergen | 0 | 1 | **1** |
| | | | | |
