# Supplementary material for: Towards Defining Molecular Determinants Recognized by Adaptive Immunity in Allergic Disease: An Inventory of the Available Data
Source: J Allergy (Cairo). 2011 Feb 13;2010:628026. doi: 10.1155/2010/628026 (PMC3042621; doi:10.1155/2010/628026)
Supplement: Supplementary file 4 [file 628026.f4.pdf]

Supplementary Table 3. Epitope Distribution in Different Airborne Allergens

	Name	T cell	B cell	Total Epitopes
**Common Airborne Allergens**				
Aspergillus fumigatus	Major allergen Asp f 2	6	93	**99**
	Ribonuclease mitogillin	8	5	**13**
	Allergen	12	0	**12**
	Oryzin precursor	0	9	**9**
	Allergen I/a; Asp f I/a	1	3	**4**
	Cellular serine proteinase	0	1	**1**
	Major allergen I	0	1	**1**
	Major allergen Asp f 1	0	1	**1**
	Allergen Asp f 4	0	1	**1**
	Superoxide dismutase	0	1	**1**
Timothy grass	Phl p 1	84	40	**124**
	Phl p 5	60	1	**61**
	Phl p 3	10	0	**10**
	Phl p 4	9	0	**9**
	Phl p 2	7	1	**8**
	Phl p 12 (Profilin-2/4)	1	5	**6**
	Phl p 13 (Polygalacturonase)	5	0	**5**
	Phl p 7 (Polcalcin)	0	2	**2**
	Phl p 11	1	0	**1**
Silver birch	Bet v 1	175	16	**191**
	Profilin (Bet v 2)	0	18	**18**
	Bet v 4	0	2	**2**
Annual ragweed	Amb a 3	4	9	**13**
	Amb a 5	8	0	**8**
	Amb a 2	1	2	**3**
	Amb a 1.1	1	1	**2**
	Amb a 1.4	0	1	**1**
Japanese cedar	Cry j 1	27	2	**29**
	Cry j IB	11	0	**11**
	Cry j 2	5	0	**5**
	Polygalacturonase	77	9	**86**
	Sugi basic protein	60	15	**75**
Perennial ryegrass	Lol p 1	46	13	**59**
	Lol p 2-A	0	2	**2**
	Lol p 3	0	1	**1**
	Lol p VA	23	20	**43**
Bermuda grass	Cyn d 1	23	1	**24**
	Cyn d 1 isoallergen isoform 1	0	1	**1**
	Cyn d 1 isoallergen isoform 3	0	1	**1**
American House dust mite	Der f 1	22	0	**22**
	Der f 2	8	9	**17**
	Allergen Mag	3	3	**6**
	Mag3	3	0	**3**
European House dust mite	Der p 2	56	32	**88**
	Der p 1	46	22	**68**
Spreading pellitory	Probable non-spec lipid-transfer protein 2	0	35	**35**
	Probable non-spec lipid-transfer protein	1	25	**26**
	Par j	0	1	**1**
Ashe juniper	Jun a 1	0	10	**10**
	Pathogenesis-related protein	0	3	**3**
Japanese cypress	Polygalacturonase	34	0	**34**
	Cha o 1	28	1	**29**

Olive tree	Ole e 1	14	39	**53**
	Beta-1,3-glucanase-like protein	0	26	**26**
Cat	Fel d 1 (Major allergen I)	48	18	**66**
Dog	Can f 1	50	0	**50**
Horse	Equ c 1	43	1	**44**
Midge	Globin Ctt 3-1	3	2	**5**
**Other Plant Species**				
Mouse ear cress	Putative pectate lyase 17	1	0	**1**
	Probable pectate lyase 18	1	0	**1**
Common velvet grass	Group V allergen	0	8	**8**
	Hol l 1	0	6	**6**
Cereal rye	Sec1	0	49	**49**
	Gamma-70 secalin isoform P4-12	0	1	**1**
Tobacco	Pectate lyase	0	1	**1**
	Cyclin-dependent kinase B1-1	0	1	**1**
Kentucky bluegrass	Pollen allergen KBG 60	18	34	**52**
Mugwort	Art v 1	19	0	**19**
Sunflower	Profilin	0	18	**18**
Great ragweed	Amb t 5	6	0	**6**
Loblolly pine	Allergen	0	4	**4**
Lichwort	Par j	0	2	**2**
Arizona cypress	Cup a 1	0	1	**1**
Formosan juniper	Allergen	0	1	**1**
Tall fescue	FeS e I	0	1	**1**
Carrot	Dau c 1	1	0	**1**
Elegant zinnia	Pectate lyase	1	0	**1**
**Other Fungal Species**				
P. brasiliensis	43 kDa secreted glycoprotein	11	0	**11**
	Immunodominant antigen Gp43	5	1	**6**
	Immunodominant antigen Gp43	0	1	**1**
Penicillium chrysogenum	Alkaline serine protease	0	30	**30**
	Pen n 18	0	15	**15**
Alternaria alternata	Major allergen Alt a 1	0	5	**5**
Malassezia sympodialis	Manganese superoxide dismutase	0	1	**1**
Candida albicans	Enolase 1	0	1	**1**
Aspergillus restrictus	Ribonuclease mitogillin p	0	1	**1**
**Other Animal Species**				
	Major urinary protein precursor	19	0	**19**
Rat	Ig epsilon chain C region	0	2	**2**
	Cytochrome P450 3A1	0	2	**2**
Storage mite	Blo t 5	0	17	**17**
	Paramyosin	0	1	**1**
Fodder mite	Lep d 2	10	5	**15**
	Allergen Cr-PI	0	4	**4**
	Cr-PII allergen (Per a 1)	0	2	**2**
American cockroach	Cr-PII allergen	0	1	**1**
	Per a 4 allergen variant 1	0	1	**1**
	Cr-PII protein	0	1	**1**
	Bla g 5	15	0	**15**
German cockroach	Bla g 4	0	8	**8**

	Bla g 2	0	2	**2**
Cow dander	Bos d 2	8	2	**10**
Mayne’s house dust mite	Eur m 1	9	0	**9**
Mouse	Naca	0	4	**4**
