# Supplementary material for: Towards Defining Molecular Determinants Recognized by Adaptive Immunity in Allergic Disease: An Inventory of the Available Data
Source: J Allergy (Cairo). 2011 Feb 13;2010:628026. doi: 10.1155/2010/628026 (PMC3042621; doi:10.1155/2010/628026)
Supplement: Supplementary file 5 [file 628026.f5.pdf]

Supplementary Table 4. Epitope Distribution Among Stinging Insects

Organism Name	Name	T cell	B cell	Overall
Honey bee	Phospholipase A2	47	4	**51**
	Melittin (Api m III)	4	7	**11**
	Apamine	0	1	**1**
	Hyaluronoglucosaminidase	0	1	**1**
Common wasp	Venom allergen 5	36	0	**36**
Bald-faced hornet	Venom allergen 5.01	20	11	**31**
Jack jumper ant	Major allergen Myr p 1	0	2	**2**
Black-bellied hornet	Mastoparan-B	0	1	**1**
