# Supplementary material for: Towards Defining Molecular Determinants Recognized by Adaptive Immunity in Allergic Disease: An Inventory of the Available Data
Source: J Allergy (Cairo). 2011 Feb 13;2010:628026. doi: 10.1155/2010/628026 (PMC3042621; doi:10.1155/2010/628026)
Supplement: Supplementary file 6 [file 628026.f6.pdf]

Supplementary Table 5. Epitope Distribution for Latex Allergens

Organism Name	Name	T cell	B cell	Overall
Latex	Pro-hevein	9	87	**96**
	Major latex allergen (Hev b 5)	15	39	**54**
	Rubber elongation factor protein	9	18	**27**
	Small rubber particle protein	10	11	**21**
	beta-1,3-glucanase	0	9	**9**
