# Supplementary material for: Towards Defining Molecular Determinants Recognized by Adaptive Immunity in Allergic Disease: An Inventory of the Available Data
Source: J Allergy (Cairo). 2011 Feb 13;2010:628026. doi: 10.1155/2010/628026 (PMC3042621; doi:10.1155/2010/628026)
Supplement: Supplementary file 7 [file 628026.f7.pdf]

Supplementary Table 6. Carbohydrate epitopes associated with allergic reactions.

Carbohydrate/Sugar Name	Source and Allergen	Category	Host	Response
α-1,3-fucose	Honey bee venom glycoprotein	Stinging Insect	Rabbit	IgG
N(4)-[α-L-fucosyl-(1->3)-N-acetyl-4-O-glycosyl-D-glucosaminyl]-L-asparagine residue	Honey bee venom phospholipase A2	Stinging Insect	Human	IgE
α-D-fucose	Celery glycoprotein	Food	Human	IgE
Fuc-α-(1->3)-[Man-α-(1->6)-[Xyl-β-(1->2)]-Man-β-(1->4)-GlcNAc-β-(1->4)]-GlcNAc	Celery glycoprotein	Food	Human	IgE
methyl β-D-glucuronoside	Beet arabinogalactan	Food	Mouse	mAb
β-D-GlcpA-(1->3)-α-D-GalpA-(1->2)-L-Rha	Beet arabinogalactan	Food	Mouse	mAb
tetragalactose	Beet arabinogalactan	Food	Mouse	IgG2c
α-L-Fuc-(1->3)-[α-D-Man-(1->3)-[α-D-Man-(1->6)]-[β-D-Xyl-(1->2)]-β-D-Man-(1->4)-β-D-GlcNAc-(1->4)]-D-GlcNAc	Cedar pollen Cry j 1	Airborne	Mouse	IgG2c
Mono-β-arabinofuranose	Mugwort pollen Art v 1	Airborne	Human	IgE
D-glucopyranuronic acid	Fungus polysaccharide	Airborne	BALB/c, Rabbit	mAb, Sera
α-D-galactosyl-(1->3)-D-galactose (α-gal)	Beef	Food	Human	IgE
xylitol	Food additive	Food	Rabbit	PCA IgE
D-GalNAc-(1->3)-[D-GalNAc-(1->4)]-D-GalNAc-(1->3)-D-GalNAc-ol	Sea Squirt H antigen	Food	Human	PCA IgE
D-GalNAc-(1->3)-[D-GalNAc-(1->2)-L-Fuc-(1->4)]-D-GlcNAc-(1->3)-D-GalNAc-ol	Sea Squirt H antigen	Food	Human	PCA IgE
D-GalNAc-(1->4)-D-GlcNAc-(1->3)-[D-GalNAc-(1->4)-D-GlcNAc-(1->6)]-D-GalNAc-ol	Sea Squirt H antigen	Food	Human	PCA IgE
D-GalNAc-(1->4)-D-GlcNAc-(1->6)-[D-GalNAc-(1->4)-[L-Fuc-(1->3)]-D-GlcNAc-(1->3)]-D-GalNAc-ol	Sea Squirt H antigen	Food	Human	PCA IgE
D-GalNAc-(1->4)-[L-Fuc-(1->3)]D-GlcNAc-(1->3)-[D-GalNAc-(1->4)-[L-Fuc-(1->3)]-D-GlcNAc-(1->6)]-D-GalNAc-ol	Sea Squirt H antigen	Food	Human	PCA IgE
